# Research hotspots and future trends of hot corrosion research: a bibliometric analysis

**DOI:** 10.1039/d3ra04628a

**Published:** 2023-10-13

**Authors:** Andrieanto Nurrochman, Endro Junianto, Akhmad Ardian Korda, Budi Prawara, Eddy Agus Basuki

**Affiliations:** a Metallurgical Engineering Research Group, Faculty of Mining and Petroleum Engineering, Institut Teknologi Bandung Jl. Ganesha, 10 Bandung 40132 Jawa Barat Indonesia; b Research Center for Advanced Material, National Research and Innovation Agency (BRIN) Serpong 15314 Indonesia budi029@brin.go.id

## Abstract

Hot corrosion has attracted researchers due to its complexity of mechanisms leading to a critical challenge for energy efficiency advancement. Literature on hot corrosion spans a wide range of discussions in materials, including metals or non-metals and operating environmental conditions. Hence it was difficult to overshadow the current status and future trends of hot corrosion research. Here we pioneered a bibliometric analysis to identify the research hotspot and possible anticipated future direction of the hot corrosion study. The results showed that at least six research hotspots can be derived after carefully classifying hot corrosion research literature based on their discussion and key findings. Some hotspots were inactive in recent years and brought complications in research direction prediction. Nevertheless, several future trends of hot corrosion research are suggested. This study provides beneficial ideas in enlightening hot corrosion research development.

## Abstract 2

Knowledge map of hot corrosion research: six research hotspots of the hot corrosion research field were elucidated.

## Graphical abstract

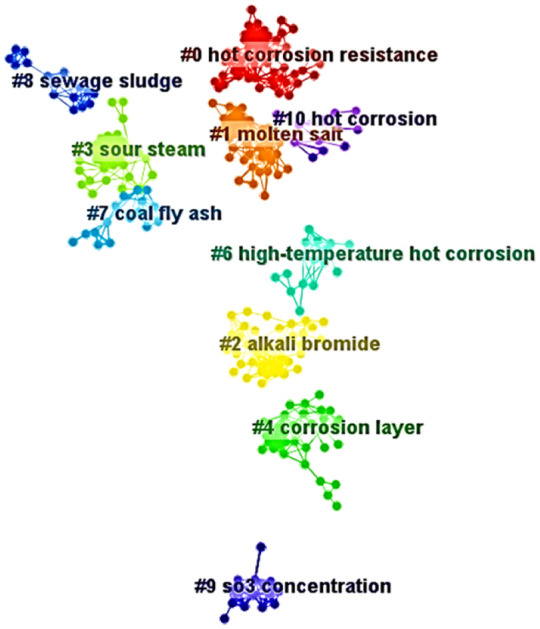

## 1 Introduction

Hot corrosion is a serious problem in hot components, especially in fossil-fueled power plants. Because in addition to the organic material that produces heat energy, fuels used for steam generation contain a wide range of impurities in the form of inorganic material.^1^ Hot corrosion occurs as a chemical reaction between those impurities of salts, chlorides, sulfides, and vanadates combined with a high-temperature environment.^2^ When subjected to high temperatures, these impurities will transform into a porous non-protective oxide scale, allowing the aggressive species to penetrate the metal surface and catalyze a rapid structure degradation process.

Two types of hot corrosion are categorized by their occurrence temperature: type I and type II.^3–8^ A type I hot corrosion, also known as high temperature hot corrosion, the attack happens at a temperature range of 800–900 °C and occurs in two stages: first, there is an incubation period during which the rate of attack is moderate as the oxide layer grows. Second is the rapid acceleration of the corrosion rate. Type II corrosion, also known as low temperature hot corrosion, occurs at temperatures ranging from 670 °C to 800 °C and is characterized by a pitting attack that is accompanied by a relatively little attack beneath the surface.

Various coating strategies have been utilized to lengthen the service life of metal components subjected to high temperatures, thereby mitigating the effects of oxidation and hot-corrosion-related degradations. Applying a well adhered coating to the alloy surface with a material that can withstand high temperatures, such as metal coating, is one of the effective ways to enhance the type I hot corrosion resistance of alloys. Though, the corrosion resistance is still not up to par.^9,10^ Another way is to introduce ceramic-type coating, which has high chemical stability. This approach can effectively separate the high temperature gas from the components, keeping the alloy safe by lowering its surface temperature.^11–13^ However, because of the vast difference in thermal expansion coefficient between the coating and the substrate, they easily peel off in cold and hot cycles.^12–15^ Therefore, it is essential to make the combination of those materials to make multi material thermal barrier coatings (TBCs).

Two ceramic and two metallic layers make up the typical TBCs structure, each bringing its unique set of thermal, mechanical, and physical properties. Some reviews from the materials design to the failure mechanisms have been done to achieve a more satisfactory performance of TBCs against destructive type I hot corrosion.^16–22^ Components coated with TBCs must be able to withstand intense heat, fluctuating temperatures, and mechanical strains. For example, industrial gas turbine engines are expected to last up to 30 000 hours of operation. The integrity of TBC during thermal exposure including hot corrosion and oxidation resistance is still a significant challenge to tackle.^23–25^ Thus, the future direction of this research is needed.

Meanwhile, aluminide diffusion coatings that can provide an alumina protective layer have been introduced and are believed to improve type II hot corrosion resistance.^26–28^ Dense alumina is widely known for effectively protecting the inward diffusion of aggressive elements, preventing the substrate from the detrimental effect of hot corrosion.^29–32^ Despite the fact that other coating elements like Cr and Co may contribute significantly to the improvement of hot corrosion resistance.^33^ Works of literature review on type II hot corrosion mechanisms and the development of protection methods have been done extensively. Patel *et al.*,^34^ in their comprehensive review article, compared the hot corrosion behavior of superalloys in four different molten salts: fluoride salts, chloride salts, nitrate salts, and sulfate salts. Nielsen *et al.*^35^ exploited the effects of chlorine on the high-temperature corrosion of biomass-fired boilers in their article. Sidhu *et al.*^36^ published an article discussing the role of high-velocity oxy-fuel (HVOF) coatings in reducing hot corrosion rate. Singh *et al.*^37^ provided an overview of plasma spray technology in depositing various coatings resistant to oxidation and hot corrosion. Recently, Shanshan Hu *et al.*^38^ thoroughly reviewed the molten salt hot corrosion test results.

Nevertheless, many previous reviews on hot corrosion were done qualitatively and mainly based on subjective understanding. Consequently, it is challenging to derive hot corrosion research hotspots and anticipated future trends from the existing published review articles. Since hot corrosion is one of the crucial issues in achieving high energy efficiency technologies,^39^ it urgently needs to conduct an in-depth analysis of its research status, hotspot, and anticipated future trend. To bridge the gap, here we report a study of hot corrosion research development through bibliometric analysis.

Bibliometric is a statistical and quantitative analysis method that studies the systematics and characteristics of the published scientific articles, books, or chapters of a book, with the purpose of better understanding the development of research in a particular field.^40,41^ This paper aims to perform a comprehensive and in-depth review of research in the field of hot corrosion through bibliometric analysis. Specifically, the objectives of this review are as follows: describe the current status of hot corrosion research from the perspective of published literature and citation outlook; map the intellectual landscape of the hot corrosion research based on the perspective of literature citations and keywords to reveal research hotspots, front and direction tendencies of research about the hot corrosion.

## 2 Methodology

### 2.1 Database search

In performing bibliometric analysis, a database of research literature is needed to select literature documents related to the hot corrosion topic. Among commonly used databases such as Google Scholar, Scopus, and ISI Web of Science (WoS), the Scopus database collection is the preferred literature data source to be adopted. Systematic and comprehensive searches were performed in Scopus database to identify publications. The search strategy for cultivating the selected literature from the Scopus database is listed in Table 1. The literature comes from the research publications from 2012 through 2021 to cover the intellectual base of hot corrosion topic. In addition, the ten years of publication were chosen because the literature published within this period has sufficient citations and is reliable for statistical analysis. The accumulated citations are relatively higher than the rate of gaining new citations in a narrower period. Thus, it can show a stable distribution state of literature citations.^42^ The “hot corrosion” phrase was utilized as a keyword to search Scopus database. Eventually, the search results with the parameters listed in Table 1 can yield 1385 published research literature for later bibliometric analysis.

**Table 1:** Literature data source search strategy

Data base	Scopus collection
Search method	Article title, abstract, keywords
Search subject	“Hot corrosion”
Time range	2012–2021
Subject area	Materials science; engineering; physics and astronomy; chemistry; chemical engineering; energy; environmental science; earth and planetary sciences
Document type	Article; conference paper; review; conference review; book chapter
Search quantity	1385

### 2.2 Bibliometric analysis

CiteSpace 6.1 was utilized in the current study to evaluate and visualize research maps and trends on hot corrosion issues. CiteSpace 6.1 is a free Java base application used to analyze and visualize co-citation networks. CiteSpace's science mapping approach can help in determining the nature of the research front, finding new trends, and detecting abrupt shifts.^43–45^ These are the essential issues that need to be addressed diligently in doing bibliometric analysis.

Price pioneered the concept of the research front.^46^ According to Price, scientists are preferred to cite most recent literature. As a result, a research front in a particular field refers to actively cited literature that mainly originates from recently published literature. A research front can represent the state-of-the-art of a certain research field. However, it is not easy to define when it began and how it has evolved as a function of time. Correspondingly, there is a critical need to recognize and assess emerging trends and abrupt changes associated with a research front across time.^47^ Furthermore, recognizing the focus of a research front at a specific period in the context of its intellectual base can help illuminate the significance of intellectual turning points as a research front evolves.

A burst detection algorithm based on Kleinberg^48^ defines an emergent research front in CiteSpace utilizing terms collected from the title, abstract, descriptor, and identifier of bibliographic entries over time. These terms are then employed as cluster labels in a heterogeneous network of terms and articles. An effective cluster labeling method based on burst-detection algorithms should be able to identify emerging trends and abrupt changes. Such a clustering approach has been successfully convinced by discovering temporal trends in the CiteSeer document database.^49^ The process sequence generally begins with determining the influential articles based on citations as the cluster's core. The cluster is then occupied with the articles co-cited with the core article. Citing articles create a research front, whereas cited articles build an intellectual base represented by co-citation clusters.

CiteSpace main user interface consists of a few panels. Few configurations were needed to conduct the bibliometrics analysis. These configurations included text source, node type, and selection criteria. Here, text sources were title, abstract, author keywords, and author keywords-plus without any specific term type. The preferred node type was reference to extract the cited references. The selection criteria were kept the default with the *g*-index and a scaling factor *k* of 25.

**Fig. 1:**
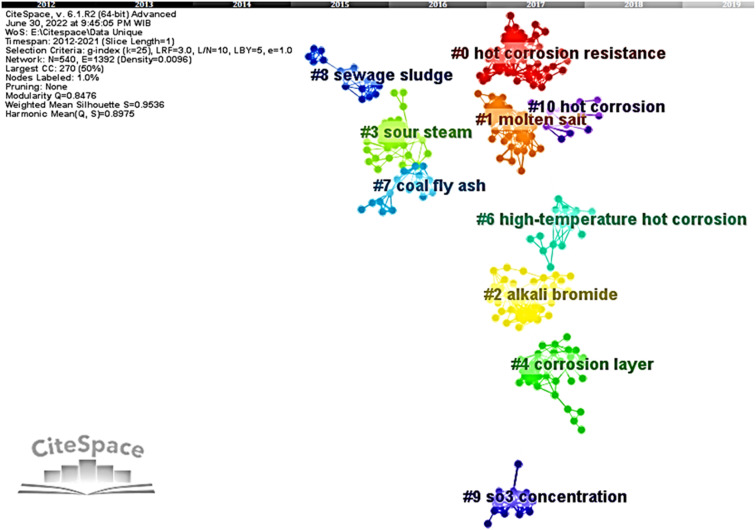
Visualization of literature co-cited network.

A map of the co-cited network by clustering was then developed and can be seen in Fig. 1. The nodes represent the references cited by citing articles, and links between them describe their co-occurrences strength. Clusters were depicted by their unique colors and automatically assigned cluster labels. Every cluster label was generated according to extraction results from keywords of citing articles and numbered based on their citation frequency. The largest cluster, which has most citation frequency, starts numbered with #0 in red and is followed by smaller clusters onwards with their unique color. The cluster relationship can be interpreted through the modularity *Q* value. Generally, the module value is between 0 and 1. The closer relationship within a cluster showed by the modularity *Q* value close to 1. However, the closer the value gets to 1, the looser relationship between clusters. Additionally, the mean silhouette *S* value informs the quality of a clustering configuration. Likewise, the closer this value is to 1, the higher the consistency of the corresponding cluster configuration. Here, modularity *Q* is 0.8476, and mean silhouette *S* is 0.9536.

Cluster labels in Fig. 1 were generated automatically based on the most representative term using the log-likelihood ratio (LLR). This algorithm was used to assign each type of label that resembled each cluster's foundation concept with scientific terms.^50^ However, clusters' labels vary due to different algorithms that can be chosen in CiteSpace. There are three algorithms in CiteSpace, including term frequency and inversed document frequency (LSI), log-likelihood ratio (LLR), and mutual information (MI). The top three terms of those different algorithms are presented in Table 2 in order to enhance the framework of interpretation. However, clustering labels extracted by CiteSpace are pretty objective and more specific than most authors' empirical driven topics or keywords. Thus, automatically derived clusters labeled by CiteSpace hardly describe research hotspots quite well. In this work, we integrate automatically clustering labels with careful readings and reviewing the article members to determine several hotspots, as shown in Table 2.

**Table 2:** Top three terms from different labeling algorithms

Hotspot ID	Hotspot topic	Cluster ID	Size	Silhouette	Mean year	(LSI)	(LLR)	(MI)
(A)	Development of TBC to improve the hot corrosion resistance against molten salts	0	51	0.6410	2016	Hot corrosion resistance; dotted coating; Al deposition	Hot corrosion resistance; dotted coating; corrosive salt	FE-SEM cross-section analysis; single step co-precipitation technique
		1	40	0.6688	2018	Sc_2_O_3_ doped ZrV_2_O_7_; 2GYTZ coating; LaPO_4_ coating; YSZ DCL TBC	Molten salt; Techna group; corrosion product	Net-like crack; induced stress; Y_2_O_3_–ZrO_2_ thermal barrier coating
(B)	Development of materials and metal coatings to improve the hot corrosion resistance against molten salts and chloride containing molten salts	2	38	0.6500	2019	Alkali bromide; power plant; anticorrosion coating	Alkali bromide; anticorrosion coating; power plant	FE-SEM cross-section analysis; Na K Al; Na_2_So_4_–NaCl molten salt
		6	16	0.6674	2020	Prolonged duration; Cr-rich carbide; hot corrosion damage	High-temperature hot corrosion; HTHC test; aggressive environment	Na_2_SO_4_–NaCl molten salt; chromium-oxide growth; tensile stress
		10	12	0.0681	2016	Aluminized NiCrAlYSi coating; Al–Si coating; Ta content	Hot corrosion; aluminized NiCrAlYSi coating; Al–Si coating	Deleterious laves phase; super alloy; arc welding
(C)	Development of materials and metal coatings to improve the hot corrosion resistance against chloride containing molten salts with the flue gas	3	34	0.6639	2016	Sour steam; alkali-induced slagging; silicate melt-induced slagging	Sour steam; alkali-induced slagging; flue gas	FE-SEM cross-section analysis; Na K Al; Na_2_So_4_–NaCl molten salt
(D)	Flue gas	9	14	0.6840	2016	SO_3_ concentration; acid dew point; coal-fired CFB combustion condition	SO_3_ concentration; heterogeneous formation; coal-fired CFB combustion condition	Flue gas condition; SO_3_ concentration increases; controlled condensation
(E)	Deposit-induced hot corrosion	7	16	0.6889	2012	Coal fly ash; alkali metal; ash transformation	Coal fly ash; low flue gas temperature; ash transformation	Biomass plant; global result; superheaters tube
		8	15	0.6583	2014	Sewage sludge; furnace wall; municipal sewage sludge	Sewage sludge; furnace wall; potassium-lead combination	Na K Al; same trend; mitigating alkali chloride formation
(F)	Hot corrosion on water-cooled wall boiler	4	34	0.6694	2019	Corrosion layer; ash deposition; ash deposit	Corrosion layer; wall temperature; acid condensation	FE-SEM cross-section analysis; Na K Al; Na_2_SO_4_–NaCl molten salt

As explained before, CiteSpace provides a burst detection feature on the number of citations count of cited references over time. In CiteSpace, published articles with citation bursts emphasize significant increases in interest in a research field. Moreover, the dynamics of a research field can manifest by citation burst in a certain period. Therefore, articles with strong citation bursts are critical indicators of the research frontiers in the corresponding field. After mapping the co-cited network, we conducted a citation burst analysis in CiteSpace. Table 3 shows the top 25 references with strong citation bursts that can reflect the development history of the research in the hot corrosion field. The references were classified by their research hotspot to specify each topic's advancement from 2012 to 2022.

**Table 3:** Top 25 references with the strongest citation bursts

References	Year	Strength	Begin	End	2012–2021	Hotspot ID
Chen Z., 2009, Investigation of reactions between vanadium oxide and plasma-sprayed yttria-stabilized zirconia coatings	2009	3.42	2012	2013	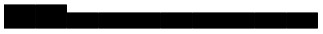	(A)
Chen Z., 2009, Degradation of plasma-sprayed yttria-stabilized zirconia coatings *via* ingress of vanadium oxide	2009	3.42	2012	2013	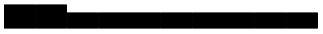	
Afrasiabi A., 2008, A comparative study on hot corrosion resistance of three types of thermal barrier coatings: YSZ, YSZ + Al_2_O_3_ and YSZ/Al_2_O_3_	2008	2.85	2012	2013	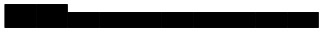	
Jiang S. M., 2010, High temperature corrosion behavior of a gradient NiCoCrAlYSi coating II: oxidation and hot corrosion	2010	2.7	2013	2014	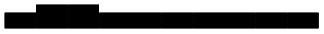	
Habibi M. H., 2012, Evolution of hot corrosion resistance of YSZ, Gd_2_Zr_2_O_7_, and Gd_2_Zr_2_O_7_ + YSZ composite thermal barrier coatings in Na_2_SO_4_ + V_2_O_5_ at 1050 °C	2012	2.66	2014	2015	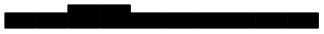	
Qiao M., 2012, Hot corrosion behavior of Co modified NiAl coating on nickel base superalloys	2012	4.5	2015	2017	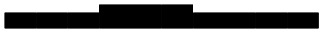	
Nejati M., 2014, Evaluation of hot corrosion behavior of CSZ, CSZ/micro Al_2_O_3_ and CSZ/nano Al_2_O_3_ plasma sprayed thermal barrier coatings	2014	3.26	2016	2017	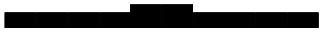	
Ajay A., 2015, Hot corrosion behavior of solution precursor and atmospheric plasma sprayed thermal barrier coatings	2015	2.99	2016	2019	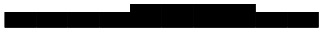	
Guo L., 2016, Comparison of hot corrosion resistance of Sm_2_Zr_2_O_7_ and (Sm_0.5_Sc_0.5_)_2_Zr_2_O_7_ ceramics in Na_2_SO_4_+V_2_O_5_ molten salt	2016	4.3	2017	2018	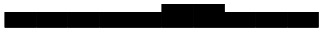	
Loghman-Estarki M. R., 2016, Comparison of hot corrosion behavior of nanostructured ScYSZ and YSZ thermal barrier coatings	2016	4.25	2017	2019	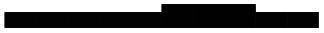	
Padture N. P., 2016, Advanced structural ceramics in aerospace propulsion	2016	2.88	2017	2021	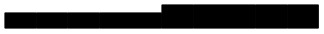	
Guo L., 2017, Hot corrosion evaluation of Gd_2_O_3_–Yb_2_O_3_ co-doped Y_2_O_3_ stabilized ZrO_2_ thermal barrier oxides exposed to Na_2_SO_4_ + V_2_O_5_ molten salt	2017	2.97	2018	2019	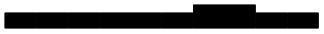	
Loghman-Estarki M. R., 2015, Evaluation of hot corrosion behavior of plasma sprayed scandia and yttria co-stabilized nanostructured thermal barrier coatings in the presence of molten sulfate and vanadate salt	2015	4.24	2019	2021	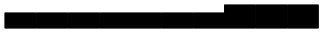	
Ozgurluk Y., 2018, Hot corrosion behavior of YSZ, Gd_2_Zr_2_O_7_ and YSZ/Gd_2_Zr_2_O_7_ thermal barrier coatings exposed to molten sulfate and vanadate salt	2018	3.83	2019	2021	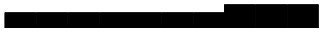	
Lortrakul P., 2014, Investigation of the mechanisms of type-II hot corrosion of superalloy	2014	3.58	2015	2018	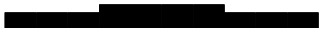	(B)
Gruber T., 2015, Investigation of the corrosion behaviour of 13CrMo4–5 for biomass fired boilers with coupled online corrosion and deposit probe measurements	2015	3.18	2015	2016	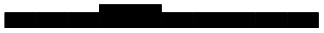	
Kleinhans U., 2018, Ash formation and deposition in coal and biomass fired combustion systems: progress and challenges in the field of ash particle sticking and rebound behavior	2018	3.29	2019	2021	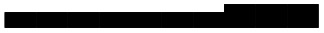	
Wang X., 2015, The ash deposition mechanism in boilers burning Zhundong coal with high contents of sodium and calcium: a study from ash evaporating to condensing	2015	2.97	2019	2021	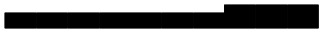	
Salehnasab B., 2016, Hot corrosion failure in the first stage nozzle of a gas turbine engine	2016	2.97	2019	2021	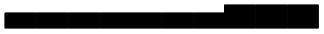	
Antunes R. A., 2013, Corrosion in biomass combustion	2013	3.86	2015	2018	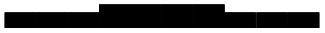	(C)
Oksa M., 2014, Nickel-based HVOF coatings promoting high temperature corrosion resistance of biomass-fired power plant boilers	2014	3.98	2017	2018	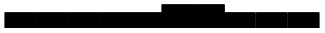	
Fleig D., 2012, Evaluation of SO_3_ measurement techniques in air and oxy-fuel combustion	2012	2.91	2016	2017	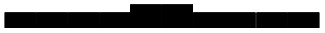	(D)
Skrifvars B.-J., 2008, Corrosion of superheater steel materials under alkali salt deposits part 1: the effect of salt deposit composition and temperature	2008	2.85	2012	2013	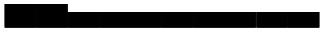	(E)
Kassman H., 2011, Measures to reduce chlorine in deposits: application in a large-scale circulating fluidized bed boiler firing biomass	2011	3.75	2013	2016	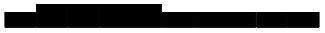	
Wang Y., 2013, Mechanism research on coupling effect between dew point corrosion and ash deposition	2013	3.09	2017	2018	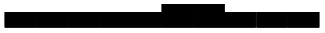	(F)

**Fig. 2:**
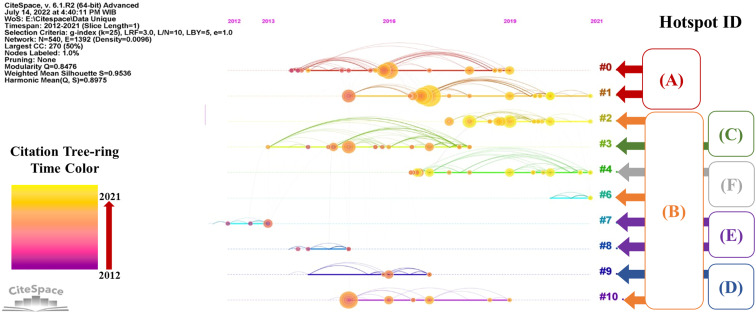
Citation relationship and research hotspot grouping in two dimensional timeline view.

The timeline perspective of the clustering diagram will complement the burst detection analysis. Timeline view through CiteSpace presents co-citation relationships of the cluster articles as a function of time. Fig. 2 shows two dimensional network by timeline visualization of cluster articles in the hot corrosion research field. Rows are cluster articles, and columns are periods. In this view, cluster articles are arranged horizontally based on their cluster, and the time value direction increases as it goes to the right side.

Citation tree rings in Fig. 2 (orangey circle) represent the citation history of an article. The legend at the bottom left of the figure explains the ring's color with the corresponding time of citations. The diameter of a ring resembles the number of citations in a given period. The bigger the diameter of the ring, the more significant the article's influence on the development of research in the corresponding field. In this work, we combine the hotspot ID in the timeline view to reveal each hotspot topic's development history in the hot corrosion research field. Therefore, with the citation burst analysis and timeline view, key article and research front of the corresponding research hotspot can be elucidated.

## 3 Results and discussion

### 3.1 Overview of research hotspots

The co-cited network reveals ten clusters in the hot corrosion research field. The cluster network resulted in a relatively high *Q* value of 0.8476. With such a *Q* value, it is reasonable for the network to be divided into several highly independent clusters. Likewise, it is implied that a well-structured clusters network was formed.^51–53^ Moreover, the mean silhouette *S* of the clusters network shows a value very close to 1, which is 0.9536 precisely. It corresponds to appropriately configured clustering results.^54^ Therefore, it can be concluded that this work's clusters of the co-cited network are quite credible and reliable.

The silhouette of each cluster in Table 1 presented a value of more than 0.5. It indicates that the clusters have high heterogeneity with only a few overlapping parts. Hereof, the cluster label is supposed to resemble the cluster member's major work. Nevertheless, we further analyzed the cluster members to reclassify based on topical category. The clusters were then grouped into six research hotspots and denoted with hotspots (A), (B), (C), (D), (E), and (F), as shown in Table 1. The hotspot (A) consisted of two clusters with ID #0 and #1. The articles in this hotspot mainly discuss TBC materials and their performance under molten salt exposure. The hotspot (B) contained three clusters of cluster ID #2, #6, and #10, which dominantly explore materials and coatings for chloride containing molten salt environments.

Cluster ID #3 and #9 were classified by their hotspot (C) and (D). The hotspot (C) articles mainly researched materials and coatings under the influence of an aggressive environment containing chloride salts and sulfur gasses. The hotspot (D) focuses on the flue gas in the boiler. The hotspot (E) has two clusters with ID #7 and #8. This hotspot mainly investigates the deposit that may induce hot corrosion in the boiler. Eventually, the hot corrosion on the water-cooled wall boiler is the hotspot (F) and was discussed by articles grouped in cluster ID #4. For further discussion, each research hotspot will be discussed as follows.

#### 3.1.1 Hotspot (A): development of TBC to improve the hot corrosion resistance against molten salts

This hotspot has been the focus of several significant articles in the hot corrosion research field. The hotspot is contained by the biggest cluster in the network, which is cluster ID #0. The second largest cluster of cluster ID #1 was also classified in this hotspot. The burst detection analysis in Table 3 shows that the first burst in this hotspot was filled by the two works done by Chen Z. *et al.*^55,56^ The burst lasted for two years which started in 2012 and ended in 2013. TBCs in gas turbine engines were the object of Chen's first work^55^ and aimed to elucidate the interaction between YSZ and V_2_O_5_ salt. YSZ was deposited with air plasma spray on a copper plate substrate. Subsequently, the coated materials were covered with V_2_O_5_ salt for the hot corrosion tests. As a result, ZrV_2_O_7_, monoclinic zirconia (m-ZrO_2_), and YVO_4_ were observed as the reaction products. Meanwhile, the second work of Chen^56^ mainly talks about the degradation mechanism of YSZ TBC exposed to V_2_O_5_ salt.

Scopus metrics provide the newest information about the citation number of Chen's first and second works. It was shown that 67 and 59 articles, respectively, had cited the first and second work. Most citations refer to the reaction between molten salts V_2_O_5_ and YSZ at high temperatures. The reaction follows the equations below.

1 ZrO_2_(t-ZrO_2_) + V_2_O_5(l)_ → ZrV_2_O_7_

2 Y_2_O_3_(t-ZrO_2_) + V_2_O_5(l)_ → YVO_4_

The tetragonal zirconia (t-ZrO_2_) experienced a significant decrease at 700 °C and 750 °C caused by V_2_O_5_ induced corrosion as described by the reactions (1) and (2).

Some researchers adopted Chen's perspective to explain similar phenomena, though different stabilizers were used. Some of the stabilizers that referred to Chen's work are CeO_2_–TiO_2_,^57^ Yb_2_O_3_–Gd_2_O_3_–Y_2_O_3_,^58–60^ Ta_2_O_5_,^61,62^ Yb_2_O_3_–Y_2_O_3_.^63^ Nevertheless, despite the aggressive V_2_O_5_ salt, Na_2_SO_4_ was also considered to react with YSZ and believed to have a significant contribution to the structure deterioration of YSZ, as described by the following reaction.

3 Na_2_SO_4(l)_ + V_2_O_5_ → 2NaVO_3(l)_ + SO_3(g)_

4 2NaVO_3(l)_ + (t-ZrO_2_)ZrO_2_Y_2_O_3_ → 2YVO_4_ + Na_2_O + ZrO_2_(m-ZrO_2_)

The studies in this hotspot were then continued to the alternative engineered materials. Based on Table 3, Habibi *et al.*^64^ initiated using Gd_2_Zr_2_O_7_ as the alternative to YSZ due to its superior stability and ability to accommodate defects than YSZ. The citation burst of Habibi's work started in 2014 and ended in 2015. One of the citing articles proposes another alternative for TBC material: Lanthanum titanium aluminum oxide (LaTi_2_Al_9_O_19_, LTA).^65^ LTA was believed to have appropriate thermal conductivity with better stability than YSZ. Nevertheless, compared to another alternative TBC material of magnesium hexa-aluminate (LnMgAl_11_O_19_ (LnMA, Ln = Nd, Sm, Gd)),^65,66^ LTA thermal conductivities were inferior.

Many other materials have been proposed since then. Lei Guo *et al.*^67^ developed RE (Rare Earth) based zirconate ceramic for TBC. The coating consisted of Sm_2_Zr_2_O_7_ and (Sm_0.5_Sc_0.5_)_2_Zr_2_O_7_. As the results, (Sm_0.5_Sc_0.5_)_2_Zr_2_O_7_ showed better hot corrosion resistance to Na_2_SO_4_ + V_2_O_5_ salt than Sm_2_Zr_2_O_7_. Moreover, prospective Ba_2_REAlO_5_ TBC, which is known to have excellent sintering resistance and ultralow thermal conductivity,^68^ was also tested in the Na_2_SO_4_ + V_2_O_5_ containing environment by Lei Guo *et al.*^69^ Interestingly, the continuous and dense layers formed on Ba_2_REAlO_5_ TBC after exposure. These layers limit the molten salts' further penetration and increase corrosion resistance.

Next citation bursts were dominantly shown by the articles that mainly discuss the architectural modification of TBC with a layered structure. Nejati M. *et al.*^70^ applied an overlaid Al_2_O_3_ above ceria stabilized zirconia (CSZ) as the third layer of TBC. The study was focused on the performance comparison of nanostructured and conventional Al_2_O_3_ layers under Na_2_SO_4_ and V_2_O_5_ molten salt. The nano Al_2_O_3_ layer showed better corrosion resistance due to the dense structure of Al_2_O_3_ that suppresses the penetration of molten salts. This study burst lasted from 2016 to 2017.

Furthermore, Gd_2_Zr_2_O_7_ was developed as the top coat above the YSZ layer of TBC in the study by Ozgurluk Y. *et al.*^71^ This study was a burst in 2019 and 2021 based on Table 3. In Orzgurluk's work, double coating layers of YSZ/Gd_2_Zr_2_O_7_ proved highly resistant to hot corrosion under molten Na_2_SO_4_ and V_2_O_5_ salts. Another double layer of TBC with LaPO_4_ as the top coat above the YSZ layer was proposed by Zhang C. *et al.*^72^ The hot corrosion tests suggested that La(P,V)O_4_ solid solution, which is less detrimental to the TBC structure. In addition, the work by Praveen K. *et al.*^73^ produced another double layer TBC with gadolinium oxide doped lanthanum cerate ((La_0.9_Gd_0.1_)_2_Ce_2_O_7_, Gd-LC) as the top coat. The promising performance of (La_0.9_Gd_0.1_)_2_Ce_2_O_7_ has then been suggested. Since, the infiltration of molten salts was inhibited.

**Fig. 3:**
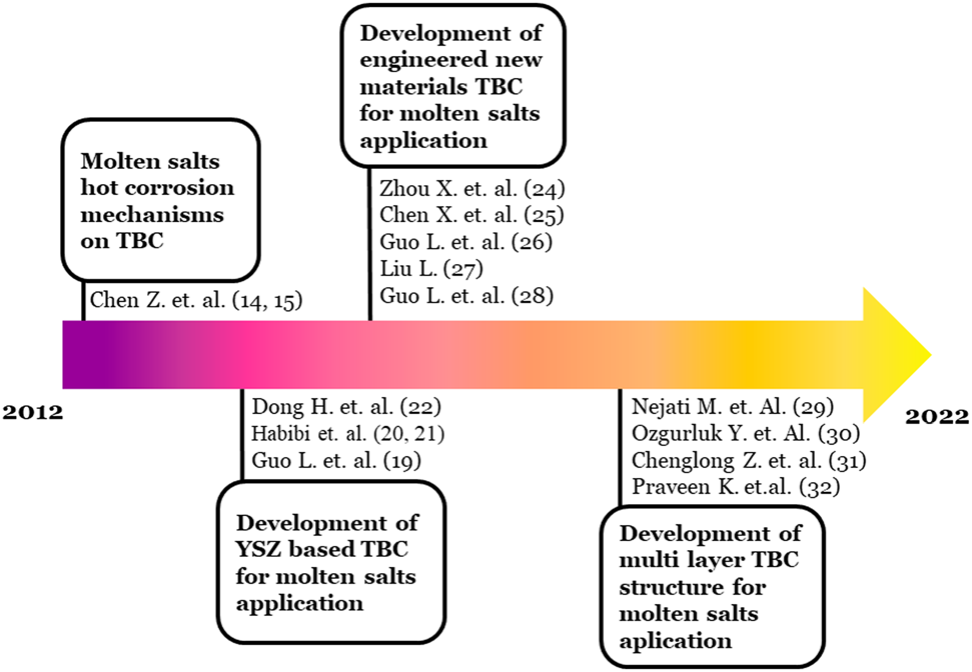
Schematic development of hotspot (A) topic of discussion.

Schematically, hotspot (A) evolution based on the burst citation analysis is shown in Fig. 3. They started with the molten salts induced hot corrosion mechanisms in the early year of the time span, to the recent active topic of TBC structure architectural modification. The references embedded in Fig. 3 were the intellectual base derived from the citation burst analysis.

#### 3.1.2 Hotspot (B): development of materials and metal coatings to improve the hot corrosion resistance against molten salts and chloride containing molten salts

In this hotspot, three clusters were classified. There were cluster ID# 2, #6, and #10. The first burst resulted from the work of Lortrakul P. *et al.*^74^ The burst of Lortrakul's work lasted for about four years which was started in 2015 and ended in 2018. Lortrakul's work focused on hot corrosion type II with a temperature range of ∼600–750 °C. The single crystal Ni-based superalloy CMSX-4 was the material being investigated. The corrosion study was conducted with a single salt deposit of Na_2_SO_4_ at 700 °C. The atmosphere gasses in the study were controlled by O_2_–SO_2_–SO_3_.

The hot corrosion mechanisms proposed in the study occurred in two stages. The first stage happened in the early hours of exposure. In the first stage, the alloy was rapidly dissolute by molten eutectic of Na_2_SO_4_–MSO_4_ (M = Ni/Co). Afterward, the second stage occurred when the MSO_4_ (M = Ni/Co) disappeared. The products of the second stage of hot corrosion were oxides and sulfides of Ni, Co, Cr, and Al.

Lortrakul's work was cited in 59 documents as recorded by Scopus. Most of the citing articles were discussed concerning the mechanisms proposed by Lortrakul's work. Especially the proposed first stage of the hot corrosion mechanisms described a rapid dissolution of the material elements by molten salt eutectic.^75–78^ Aside from Lortrakul's work, the next burst was presented by the work by Gruber T. *et al.*,^79^ which lasted two years in 2015 and 2016. Compared to Lortrakul's work, the hot corrosion studied by Gruber may have a similar type of hot corrosion type II. However, Guber specifically did a study focused on the hot corrosion mechanisms in biomass boilers that can contain more aggressive salts, such as chloride containing salt. Boiler steel 13CrMo4–5 was the material being investigated. Gruber did the research in the actual biomass boiler environment. In the study, oxidation by oxygen was the main corrosion mechanism.

Nevertheless, their further work^80^ suggested a significant effect of KCl deposit on the surfaces of the materials. In the related case, Cl may induce oxidation and accelerate hot corrosion.

Several works cited by Gruber T. *et al.* tried to investigate different biomass materials that fueled boiler power plants. Xiong W. Z. *et al.*^81^ studied the 12Cr1MoV steel. While various steel alloys of X8CrNiMoVNb16–13, X8CrNiMoNb16–16, SA213-TP347HFG, and 12Cr18Ni12Ti were investigated by Dedov A. *et al.*^82^ Moreover, Liu Y. *et al.*^83^ was compared the biomass environment with high and low chlorine contained coal to reveal the hot corrosion behavior of common boiler steels T91, 12Cr1MoVG, and TP347H. The result showed that high-Cl environments were more destructive to the steel specimens.

On the other hand, the next burst that presented the work by Kleinhans U. *et al.*^84^ put effort into describing the ash formation and deposition in coal and biomass fired combustion systems. The burst of the work has lasted for three years, starting in 2019 and ended in 2021. Kleinhans's work emphasizes that chlorine in ash deposits was a significant factor in accelerating the corrosion rate of the boiler materials in coal or biomass fueled. Further, Ma W. *et al.*^85^ specifically explored the effect of chlorine during municipal solid waste (food waste, biomass, yard waste, and others) incineration. Ma W.'s work explained that most of the corrosion of the boiler tubes was induced by chlorine gas and alkali chlorides. Ma W. proposed an alumina forming nickel based alloy to lengthen the materials life when Cl is contained in the exposure environment.

The other burst was based on the hot corrosion failure experience of Salehnasab B. *et al.*^86^ In the study, a nickel based alloy of Nimonic105 was corroded due to sulfate that formed by a reaction of chloride salt with sulfur from the fuels. One recommendation was to use a coating that may increase the corrosion resistance of the materials. Accordingly, Zhang X. *et al.*^87^ work has compiled some coating materials that have been studied to combat the hot corrosion in power plants with low-grade solid fuels. According to Zhang, high velocity oxy-fuel (HVOF) was the most preferred depositing method for researchers to study the hot corrosion behavior of coatings. Nevertheless, the coatings' behavior is unique to the exposure environment, which is highly influenced by the fuel used.

In the case of coating in coal fired boiler, from the Scopus database, we searched the literature with the string “coating AND coal AND boiler” and found 97 documents. Then, we manually reclassified the literature from 2017 to 2022 to observe the advancement of high temperature coatings for coal fired boilers. The first literature showed that superalloy substrates were coated with double layers of NiCrAlY as a bond coat and Ni_3_Al as the top coat in work by Mishra S. B. *et al.*^88^ The Al_2_O_3_, which formed on the coating surface, was found as the perfect barrier against corrosion. Furthermore, some works on coal-fired boiler coating development are shown in Table 4, including their important finding relating to hot corrosion in coal fired boiler.

**Table 4:** Works on coatings development for coal-fired boiler

Deposition method	Material	Key finding	Ref.
HVOF	93(WC–Cr_3_C_2_)–7Ni	Oxides of Ni and Cr, along with WC, result in enhanced corrosion resistance	89
	86WC–10Co–4Cr		
	83WC–17Co		
	75Cr_3_C_2_–25NiCr		
Detonation gun	Cr_2_O_3_–75% Al_2_O_3_	Well adhered coating resulted, and Al_2_O_3_ and Cr_2_O_3_ were present on the surface. These oxides inhibit some oxidizing agents from penetrating	90
Detonation gun	Cr_3_C_2_–25%NiCr	The presence of Cr_2_O_3_ may be responsible for preventing hot corrosion	91
PVD	Nanostructured TiALN	The Al based oxides were formed and increased the corrosion resistance	92
Cold spray	NiCr	The continuous Cr_2_O_3_ provided satisfactory erosion-corrosion resistance	93
	NiCrTiC		
	NiCrTiCRe		
Pack cementation	Al	Fast degeneration of the Al reservoir leads to extensive growth of the Al-coating oxide. At the same time, Cr-oxide on Cr-coating has better protection against corrosion	94
	Cr		
High-volume low-pressure (HVLP) spraying	h-BN + graphite	The coating was highly dense and prevented the diffusion of aggressive species into the substrate	95
Plasma spray	70% NiCrAlY/25WC–Co + 30% cenospheres	NiO, Cr_2_O_3_, and Al_2_O_3_ oxides acted as diffusion barriers for oxygen	96
Plasma spray	Fe based amorphous	Cr_2_O_3_, NiO, and NiCr_2_O_4_ formed on the surface increase the corrosion resistance	97
HVOF	CoCrAlYTaCSi	The Cr_2_O_3_ contributed to the enhanced hot corrosion resistance	98
	Cr_3_C_2_–25%NiCr		
Wire arc spray	Ni–20Cr	The protective oxides of Cr_2_O_3_, NiO, along with NiCr_2_O_4_ were formed	99
HVAF	Ni–Cr alloy (Cr: 30 at%, 45 at%, and 50 at%)	Higher the Cr content, the better the corrosion resistance. The oxides of Cr_2_O_3_ and NiCr_2_O_4_ were the principal protective oxides formed	100
	Ni–Cr–Ti		
Detonation gun	NiCoCrAlY	The NiCr_2_O_4_ was the oxide responsible for the enhanced corrosion resistance	101
Electrodeposition	45CT	The amount of Cr in the coating is directly proportional to its resistance to corrosion because the protective Cr_2_O_3_ inner scale inhibits Ni's outward diffusion and O and S's inward diffusion	102
	NiCrMo_13_		
	NiCrBSi		
	Alloy 718		
	Alloy 276		
Plasma spray	Al_2_O_3_–0 wt% CNT	CNT filled in Al_2_O_3_ defects and improved the Al_2_O_3_ microstructure, thus increasing the corrosion resistance	103
	Al_2_O_3_–1.5 wt% CNT		
	Al_2_O_3_–2 wt% CNT		
	Al_2_O_3_–4 wt% CNT		
HVOF	Cr_3_C_2_ + NiCr + multi-walled CNT	CNT reduces the coating porosity and inhibits the diffusion of corrosive species	104
Wire arc spray	Ni–20Cr	The Cr_2_O_3_, NiO, and NiCr_2_O_4_ were the protective oxide that increased the corrosion resistance	105
	Ni–5Al		
Detonation gun	WC–Co (88–12%)	The coating acted as the sacrificed material for the substrate to protect	106

On the other hand, materials in biomass-fueled power plants pose a significant threat to chloride-containing salt deposition. In the Scopus database, 41 documents were shown with the string “coating AND biomass AND boiler” from 2017 to 2022. After carefully reviewing the literature, Table 5 shows published research concerning the coating used in the boiler with biomass fuel. Some findings are presented and may provide a substantial intellectual base for future research direction.

**Table 5:** Works on coatings development for biomass boiler

Deposition method	Material	Key finding	Ref.
HVOF	Inconel 625	A lower amount of Cr and Ni and a high amount of Fe may decrease corrosion resistance	107
	Inconel 718		
Electric arc spraying	Fe–Cr–Mo amorphous coating + sodium silicate sealant	Cl acted as a catalyst for accelerating corrosion, and Fe and Cr were mainly chlorinated	108
Air plasma spraying (APS)	Ni–Al (Al = 5 wt%, Ni = balance) + preoxidation at 700 °C for 6 h	The porosity produces poor corrosion resistance, and pre-oxidation can hinder the ingression of corrosive species	109
HVOF	Stellite-6	Adequate protection by Cr_2_O_3_ outer layer and the sub-layer rich in Co (CoCr_2_O_4_ and CoO) resulted from the Stellite-6 coating. While NiAl coating severely damaged due to a chlorine attack	110
	NiAl		
Electroplating + pack aluminizing	Ni	Nickel aluminide coating was corroded and formed AlCl_3_	111
	Ni_2_Al_3_		
HVOF	Ni_5_Al	Cr oxides were blocking inter-splat corrosion attack, thus mitigating internal corrosion	112
	Fe_50_Cr		
	Ni_20_Cr		
HVAF	Ni_21_Cr_9_Mo	SiO_2_ induces the rapid formation of dense and highly attached Cr_2_O_3_ that hinders chloride diffusion	113
	SiO_2_-containing Ni_21_Cr_9_Mo		
HVAF	Ni_21_Cr_7_Al_1_Y	Chloride reacted with Cr and Al to form CrCl_3_ and AlCl_3_ at the splat boundaries, while Mo can support the formation of a protective Cr oxide scale	114
	SiO_2_-containing Ni_21_Cr_9_Mo		
Electroplating + pack aluminizing	Ni	Cl species was diffuse and propagated the corrosion reaction	115
	Ni_2_Al_3_		
HVOF	NiAl	Inhibiting the diffusion of Cl_2_ and O_2_ is crucial because Cl_2_ acts as a catalyst, inducing the formation of NiCl_2_, AlCl_3_, and subsequent oxidation at the interface	116
HVAF	NiCr (in wt%; 78.6 Ni–21.3 Cr–0.1 O)	Cl^−^ may diffuse and form metal chlorides through defects and splat boundaries. Moreover, Al_2_O_3_ was found to impede the Cl^−^ diffusion	117
	NiAl (in wt%; 94.1 Ni–5.7 Al–0.2 O)		
HVAF	Ni_5_Al	Al-rich oxide was observed to have better protection against KCl. Cr-rich oxide was prone to degradation due to chromate formation	118
	Ni_21_Cr		
	Ni_21_Cr_7_Al_1_Y		
	Ni_21_Cr_9_Mo		

Municipal solid waste, on the other hand, is also categorized as one of the biomass resources classified as renewable.^119,120^ However, high Cl concentration poses a more destructive threat to boiler components' reliability in waste fueled power plants.^121^ Several coating approaches to attain adequate performance in a high chlorine environment have been done, as shown in Table 6. The works in Table 6 were extracted from the Scopus database from 2017 to 2022 with the search string “coating AND waste AND boiler”. It is important to note that 41 documents have resulted from the search; however, there was some overlapping literature database with the biomass fueled boiler. Thus, only few of them specifically studied the materials used in waste fueled boilers.

**Table 6:** Works on coatings development for waste fueled boiler

Deposition method	Material	Key finding	Ref.
Arc spraying	NiCrTi	The nature of the splat boundaries impeded the Cl inward diffusion in the microstructure contained by Cr and Ni rich oxides	121
	NiCrMo		
HVAF	NiCr	The Al rich oxide was able to impede the diffusion of Cl into the substrate	130
	NiAl		
	NiCrAlY		
Cold spray	Cr_3_C_2_–Ni_20_Cr	Ni and Cr based oxides were efficient in suppressing the diffusion of corrosive species into the substrate	131
Plasma transferred arc welding (PTA)	NiCr	HVOF coating method has better corrosion resistance in the test conditions. It was believed that Cr content could increase the corrosion performance of the coating	132
Twin wire arc spray (TWAS)	FeCr		
HVOF			

**Fig. 4:**
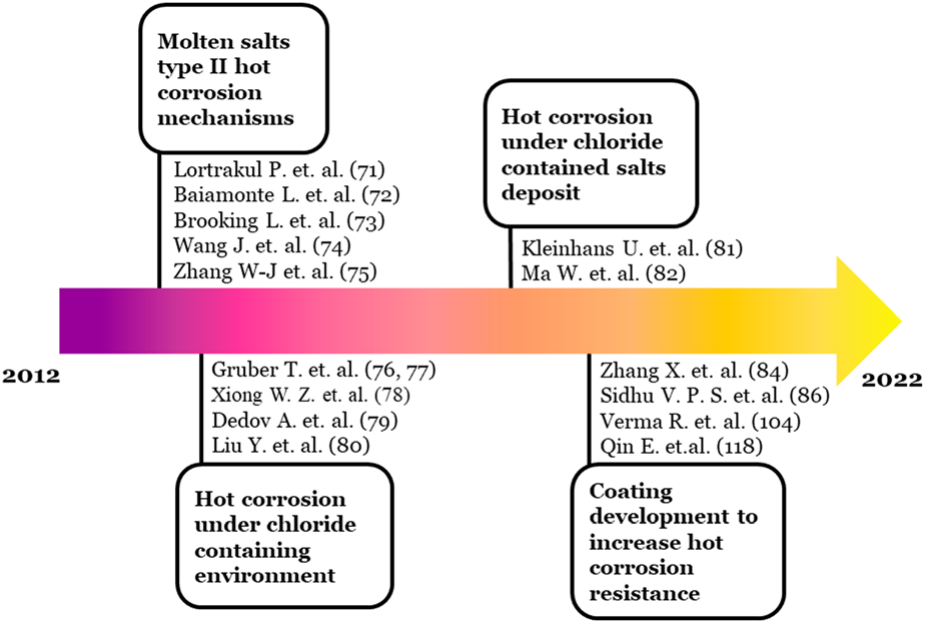
Schematic development of hotspot (B) topic of discussion.

Based on the bursts analysis of hotspot (B), the schematic development of the research in hotspot (B) is described in Fig. 4.

The earlier research in this hotspot discussed the molten salts' hot corrosion mechanisms. Several researchers classified the hot corrosion in this hotspot as mostly included in type II hot corrosion that occurs at relatively lower temperatures.^74–78^ Afterward, the effect of chloride, either as the surrounding environment or salts deposit, was the focus of several researchers. Furthermore, most of the literature in this hotspot recently worked on developing the coating materials to combat the hot corrosion in power plant boilers.

#### 3.1.3 Hotspot (C): development of materials and metal coatings to improve the hot corrosion resistance against chloride containing molten salts and the flue gas

In the hotspot (C), the member is only the cluster ID #3. Hence, the literature member of cluster ID #3 shapes the topic scope of the hotspot (C). The burst in this hotspot resulted from the work of Antunes R. A. *et al.*^122^ and Oksa M. *et al.*^123^ Antunnes *et al.* published work on the selection of materials for biomass combustion. In their work, the influence of flue gas on the corrosion mechanisms was found to be significant. Especially the SO_2_ gas that exhibited a positive effect on the corrosion rate. They proposed that the reaction between KCl and SO_2_ led to the formation of a thin and dense K_2_SO_4_ layer that can suppress the inward diffusion of chloride species such as HCl and Cl_2_. Based on this observed phenomenon, sulfur-rich fuels are better combined with chlorine-rich biomasses to prevent chloride-containing deposits.

Some authors experienced similar observations concerning the influence of SO_2_ on the hot corrosion behavior in a chlorinated environment.^124–129^ When SO_2_ exists in the atmosphere, it will react with KCl through the following equation.

5 2KCl_(s)_ + SO_2(g)_ + O_2(g)_ ↔ K_2_SO_4_ + Cl_2_

**Fig. 5:**
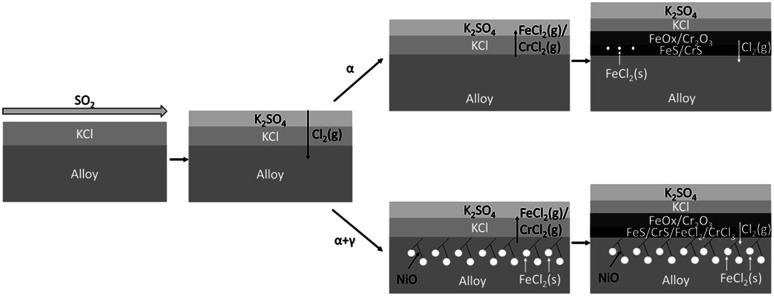
Schematic overview of the proposed corrosion mechanism depending on the microstructure.^133^

The work of Phillip *et al.*^133^ proposed the aforementioned hot corrosion mechanisms, as seen in the following Fig. 5.

KCl_(s)_ deposit will be sulfated after exposure to SO_2_-containing flue gas. The reaction in eqn (5) will take place at temperatures below 1000 °C. When the flue gas has a higher temperature, the salt deposit will be melted above the oxide scale. However, due to the formation of eutectics mixtures such as KCl–FeCl_2_, the melting point becomes 393 °C, much lower than most boiler operating temperatures.^134^ These melts can increase corrosion attack by covering the material surface with Cl contained melts that can accelerate coating elements' dissolution.

On the other hand, Oksa M. *et al.*^123^ studied the high chromium coatings performance in actual biomass power plants. HVOF deposited the coatings on carbon steel St35.8. The materials of the coatings were NiCr_16_Mo, NiCr_9_Mo, and NiCr_10_Al. The results showed that Cl's detrimental effect prevailed without coating. It was proposed that the material surface covered by the deposit melts containing Cl and accelerates the corrosion process. However, the influence of the flue gas, such as SO_2_, was not strongly detected. Great concern was developed to the high amount of water vapor in flue gases which contribute to the formation of non-protective oxides. Nevertheless, the coatings presented excellent performance during the test. A compound of Cl contained was observed next to the surfaces of the coatings; however, the corrosive species seemed inhibited from penetrating.

Overall, the hotspot (C) based on the burst analysis can be divided into two main discussions. The first main discussion is on the measure to decrease the aggressiveness of the chloride through sulfidation, additive utilization, and fuel pre-treatment. The articles with this main discussion topic are distinguished historically into two specific topics. The earlier published articles^135–137^ covered the topic about elucidating the Cl role and the occurrences of hot corrosion under Cl contained deposits. While the later published articles work on the topic of methods of controlling the Cl content in the flue gas or deposit.^138–141^

**Fig. 6:**
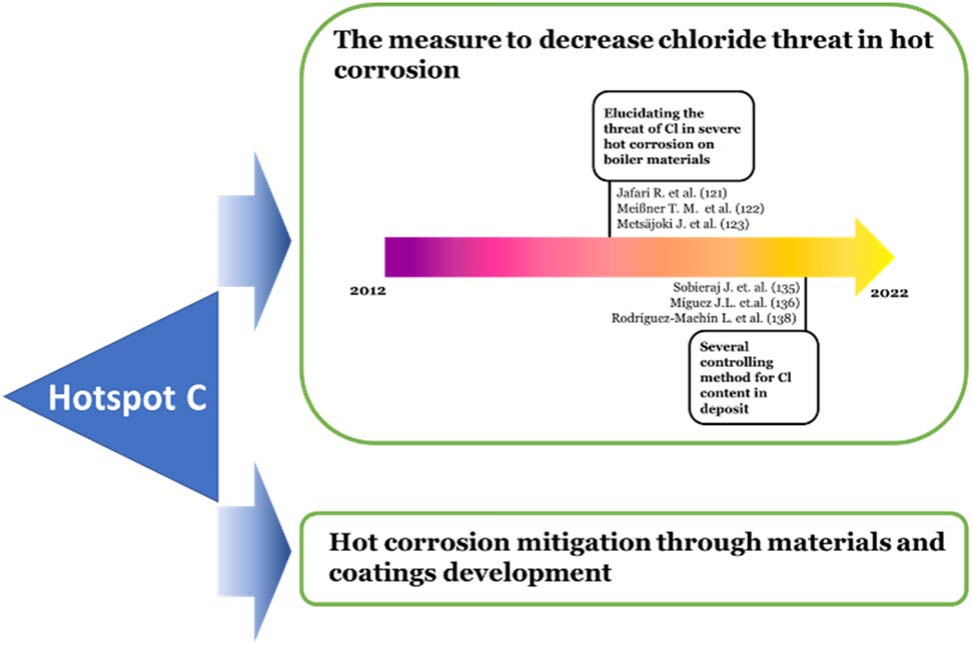
Schematic development of hotspot (C) topic of discussion.

The articles covering the second main discussion were dominated by those cited in Oksa's work and counted as 58 published articles per the Scopus database. As mentioned above, most of the works focused on the materials and improved coatings to mitigate the hot corrosion. The research development in the hotspot (C) is schematically shown in Fig. 6.

#### 3.1.4 Hotspot (D): flue gas

The member of this hotspot is cluster ID#9. The burst analysis shown by the work by Fleig D. *et al.*^142^ In their study, concern arose about evaluating SO_3_ measurement techniques in the boiler. SO_3_ formation may stimulate fireside corrosion by forming alkali iron trisulfate. Nevertheless, due to its high reactivity, SO_3_ is known to be challenging to measure. The work by Fleig D. *et al.* gave a brief review of several SO_3_ measurement techniques.

Forty-eight published works cited the burst article by Fleig D. *et al.* They mainly discussed the formation and detection of SO_2_ and SO_3_ gasses.^143–150^ In the coal-fired boiler, the sulfide source is mostly pyrite (FeS_2_).^151^ The temperature and oxygen concentration highly influence sulfide release reactions from pyrite.^152^Eqn (6)–(8) describe the reaction that may occur during sulfide release from pyrite and then SO_2_ formation.

6 2FeS_2(s)_ → 2FeS_(s)_ + S_2(g)_

7 2FeS_(l)_ → 2Fe + S_2_

8 S_2_ + 2O_2_ → 2SO_2_

At temperatures above 1000 °C and under oxygen-rich conditions, SO_2_ is preferred to be formed thermodynamically. For instance, coal containing sulfur tends to oxidize into SO_2_ at 1400 °C.

Generally, sulfur in coal is released into SO_2_ during the combustion in the boiler. Only a few of SO_2_ further oxidized into SO_3_. However, the disadvantageous effects of SO_3_ on the environment have attracted much attention.^153,154^ Several drawbacks of SO_3_ emission have been reported, such as visible plumes, acid rain, skin irritation, and corrosion.^154^ SO_3_ is formed through either a homogeneous gas phase or heterogeneous reactions.^155^ Both reactions are presented in Table 7.

**Table 7:** SO_3_ formation reactions

Homogeneous reactions	Heterogeneous reactions
SO_2_ + OH(+M) ↔ HOSO_2_(+M)	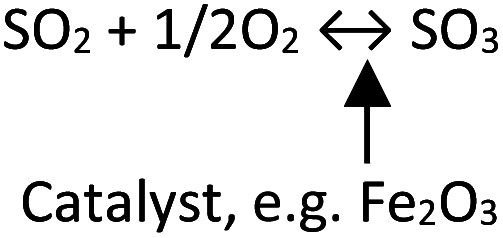
HOSO_2_ + O_2_ ↔ SO_3_ + H_2_O	
*M is the third body molecule or chaperone	

The homogeneous reactions are influenced by SO_2_ content, temperature, moisture, and other reactive gasses like NO, CO, and CH_4_. While in the heterogeneous reactions, ash concentration and composition significantly contribute as the catalytic oxidizer for SO_3_ formation.

On the other hand, in biomass-fueled boilers, reducing NOx emissions is an urgent problem in achieving a healthy environment.^156,157^ At least three reaction mechanisms have been proposed for NOx formation during combustion.^158^ First is known as the thermal NOx mechanism, which occurred due to high temperature (1300 °C) exposure. The second mechanism is called fuel NOx, which refers to fuel-N oxidation. The last is prompt NOx mechanisms due to chi-radicals reacting with nitrogen in the atmosphere.

Apart from nitrogen, biomass commonly has only a few sulfur contained. These sulfur formed SO_2_ and mostly reacted with the ash constituents, especially Ca, K, and Na forming sulfates.^156^ Other gasses that are released during biomass combustion are chlorine based gas compounds. Some of those gasses that have been observed are hydrochloric acid (HCl), potassium chloride (KCl), sodium chloride (NaCl), lead chloride (PbCl_2_), and zinc chloride (ZnCl_2_) (149 150).^159,160^ Chloride containing gasses may accelerate the hot corrosion of the boiler tube.

#### 3.1.5 Hotspot (E): deposit-induced hot corrosion

In the hotspot (E), cluster ID#7 and ID#8 are the main contributing clusters. The discussion about deposit-induced hot corrosion dominated the topic. Two articles detected by CiteSpace have significant citation bursts from the burst analysis. First is the work by Skrifvars *et al.*^161^ and the second is an article published by Kassman *et al.*^162^ In Skrifvars work, corrosion under salt deposits was discussed, and extensive laboratory testing was done. The hot corrosion test was performed in the temperature range from 450 °C to 600 °C for 168 h under an air atmosphere. The deposits were synthetic alkali salts with sodium sulfate, potassium sulfate, and sodium chloride variations. Low alloy steel, 10CrMo9–10, steel T91, Esshete 1250, and a nickel-based alloy were among the materials considered for the boiler's superheater tubing.

They proposed that the hot corrosion phenomena under deposit can be divided based on the exposure temperature. Their work found that the exposure temperature highly influenced the deposit's physical state. Accordingly, the hot corrosion might have different mechanisms at the temperature of the deposit containing melt and at the temperature below no melt in the deposit. Evidently, the hot corrosion was escalated by the presence of melt in the deposit, and chlorine found in the deposit melts was like a catalyst for hot corrosion.

Their work was cited by 127 articles based on the Scopus database. It means that other works also observed the acceleration of hot corrosion due to the existence of melts and chlorine in the deposit proposed in their study. Selected papers and their main findings are shown in Table 8. Furthermore, the citation amount may have described that the topic of deposit-induced hot corrosion has gained enormous attention.

**Table 8:** Selected citing articles focus on corrosion under deposit

Findings	Title	Year	Ref.
Cl contributes as a catalyst, which accelerates the corrosion process	Comparative study on ash deposit mechanism and characteristics of eucalyptus bark and bagasse-firing on boiler superheater	2022	163
A high concentration of Cl_2_ accelerates the oxidation of the material alkali chloride salt was found to be more aggressive than alkali sulfate salt	Elevated temperature molten salt corrosion study of SS304L austenitic boiler steel	2020	164
Cl contained salt mixture may trigger corrosion below the melting temperature, and the corrosiveness of the salt mixture increase as the Cl concentration increases in the deposit	Fireside corrosion on T24 steel pipes and HVOF NiCr coatings exposed to different salt mixtures	2020	165
Alkali chloride may form low melting temperature compounds and accelerate the hot corrosion	High-temperature corrosion performance of HVAF-sprayed NiCr, NiAl, and NiCrAlY coatings with alkali sulfate/chloride exposed to ambient air	2019	124
Hot corrosion was observed to be more aggressive under the deposit of higher alkali chlorides than alkali sulfates salt chlorides induced cracks and deteriorating oxide scale like Cr_2_O_3_	Hot corrosion study of 9Cr–1Mo boiler steel exposed to different molten salt mixtures	2018	166
The alkali sulfates salt reduces the aggressiveness of alkali chlorides	Effect of synthetic biomass ash on high temperature corrosion behavior of super austenitic stainless steel 904L	2019	167
This study observed potassium chloride (KCl) as the most corrosive species in a biomass combustion environment	The effect of oxygen source on the reaction mechanism of potassium chloride-induced high-temperature corrosion	2018	168
The defects in the corrosion layer, such as cracks and voids, were penetrated by chlorides and significantly severed the corrosion attack	Hot corrosion studies of boiler steels exposed to different molten salt mixtures at 950 °C	2019	169
The hot corrosion mechanism starts with the formation of protective oxides, which then the chlorides containing salt cause spallation or dissolution of these oxides	Hot corrosion behavior of HVOF-sprayed Cr_3_C_2_–NiCrMoNbAl coating	2017	170
Alkali sulfates alone do not cause corrosion below 500 °C	High-temperature corrosion due to lead chloride mixtures simulating fireside deposits in boilers firing recycled wood	2017	171
A eutectic mixture including FeCl_2_–KCl–PbCl_2_, which melts below 350 °C may accelerate the hot corrosion			
At temperatures beyond 450 °C, the corrosion mechanism was mainly alkali chloride-induced corrosion	The influence of flue gas temperature on lead chloride induced high temperature corrosion	2017	172
The presence of SO_2_ in the HCl atmosphere may reduce the corrosion rates. In this study, aluminum was found to have a greater tendency to form chlorides than chromium and iron	Elevated-temperature corrosion of uncoated and aluminized 9–12% Cr boiler steels beneath KCl deposit	2014	126
In the presence of SO_2_, chloride containing salt (NaCl) preferably reacts with atmospheric gasses to form Na_2_SO_4_ rather than interact with Cr or C_2_O_3_	Corrosion mechanism of alloy 310 austenitic steel beneath NaCl deposit under varying SO_2_ concentrations in an oxy-fuel combustion atmosphere	2013	173

**Fig. 7:**
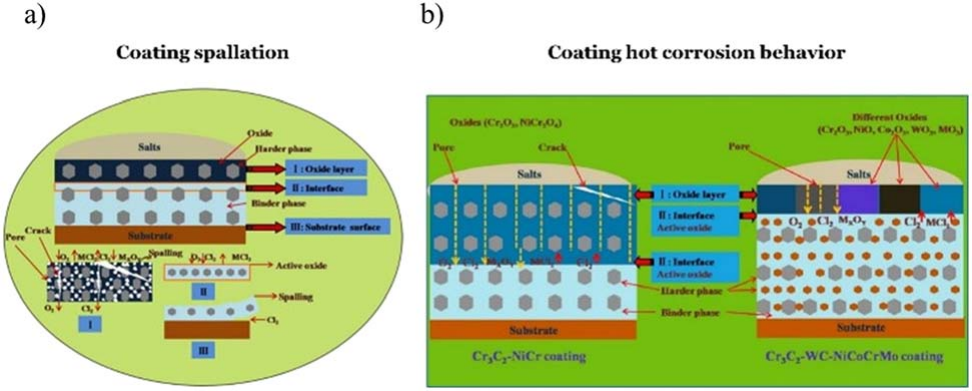
Schematic diagram of deposit induced hot corrosion mechanisms on NiCr base coating, (a) Cr_3_C_2_–NiCr coating spallation mechanisms^170^ and (b) hot corrosion behavior of Cr_3_C_2_–NiCr and Cr_3_C_2_–WC–NiCoCrMo coating.^174^

The schematic mechanisms of the deposit induced hot corrosion are presented in Fig. 7 below. At first exposure of the coating, several oxides were observed to be formed. The intrinsic defects of the coating structure, like splat boundaries and pores, let the oxygen and chlorides penetrate the coating at the subsequent stage.^174^ Several reactions of oxides dissolution and metal chloride formation were observed at the later stage. Consequently, the loose oxides were created primarily near the surface area of the coating. As the exposure time increase, the coating may be spalled (Fig. 7a). However, the deterioration of the oxide structure was possibly hindered by using some oxide forming elements as the alloying mixture (Fig. 7b).

On the other hand, the work by Kassman *et al.*^162^ was cited by 89 articles as recorded by Scopus. Their work also focused on the hot corrosion under deposit in the superheater. However, the test was conducted in the actual boiler with the deposits formed and growth accordingly influenced by the exposure temperature. They believed that chlorine in the deposit was better reduced and proposed that sulphation may limit the chlorine in the deposit.

#### 3.1.6 Hotspot (F): hot corrosion on water-cooled wall boiler

**Fig. 8:**
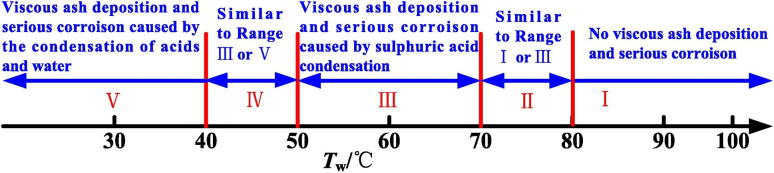
Ash deposition and corrosion characteristics as a function of wall temperatures.^173^

In hotspot (F), the strongest citation burst resulted from an article produced by Yun-Gang Wang *et al.*^175^ The exhaust part of the coal fueled boiler was the focus of their study. The research mainly discussed the corrosion at dew point, so at lower temperatures compared to most of the burst works above. In Yun-Gang Wang work, the temperature ranged from 30 °C to 80 °C. Nevertheless, the corrosion characteristic depended on the temperature and possibly differed as temperature ranges through the tube wall. Heng Chen *et al.*^176^ proposed the corrosion characteristics based on the tube wall temperature, as seen in Fig. 8.

At the dew point, the acid condenses and then reacts with the tube surface. Furthermore, condensed acid reacts with some ashes and forms viscous fouling.^177^ The dew point corrosion process in coal fueled boiler was expected to take place as the following reactions:

9 SO_3(g)_ + H_2_O_(g)_ ↔ H_2_SO_4(g)_

10 H_2_SO_4(g)_ on condensation at ADPT ↔ H_2_SO_4(l)_

11 H_2_SO_4(l)_ + M (fresh metal surface) ↔ M_*x*_SO_4(s)_ + H_2(g)_

**Fig. 9:**
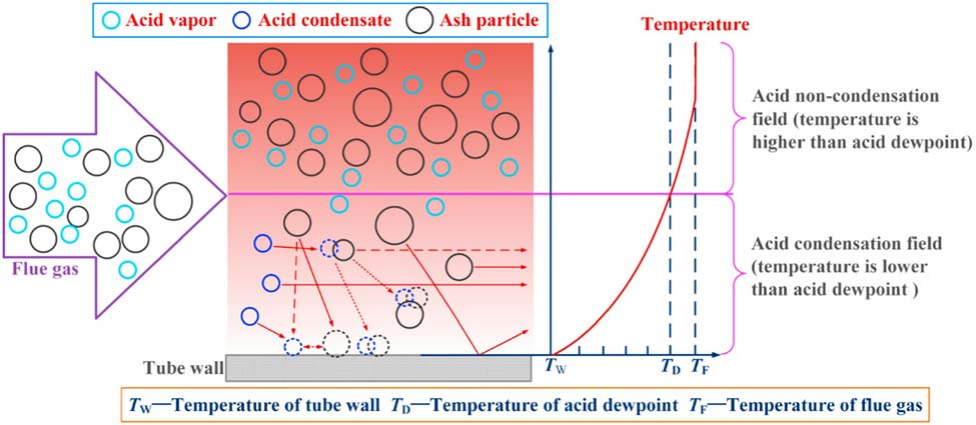
Coupling model of acid condensation and fly ash.^178^

**Fig. 10:**
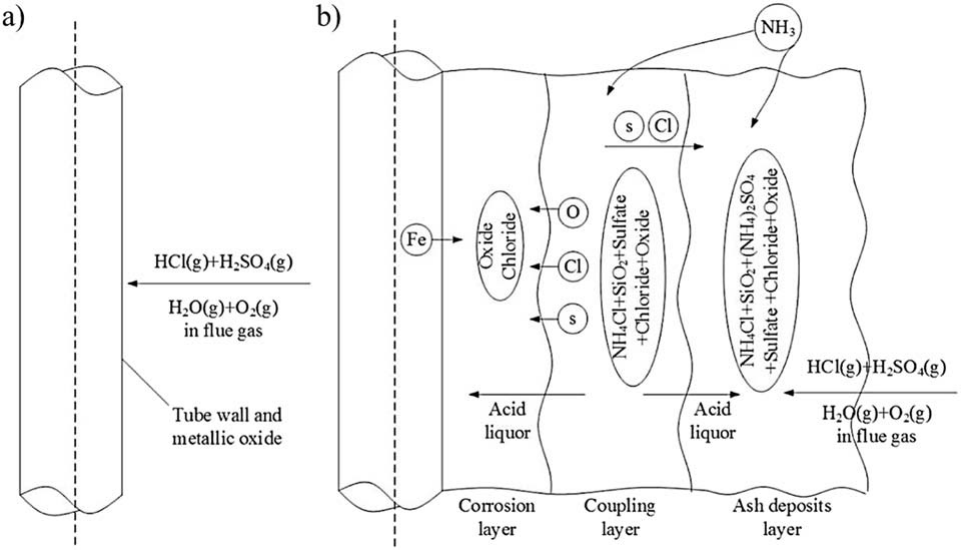
Schematic of dew point corrosion process. (a) The first stage. (b) The second stage.^179^

Schematically, the corrosion process was suggested in Heng Chen *et al.*^178^ study and shown in Fig. 9, which included the coupling effect between ash and the flue gases. In biomass fueled boilers, the majority of the ash was observed to be NH_4_Cl.^179^ The corrosion mechanism was believed to follow the schematics shown in Fig. 10.

**Fig. 11:**
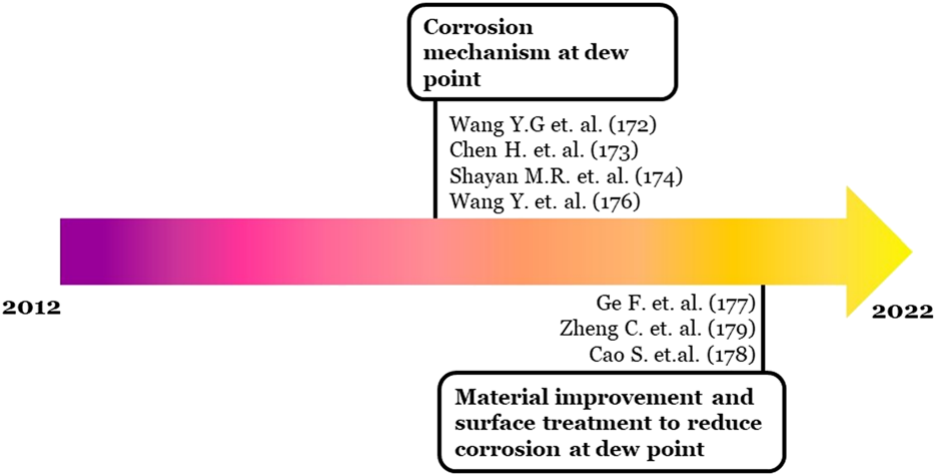
Schematic development of hotspot (F) topic of discussion.

Sixty-three documents have cited the article of Yun-Gang Wang *et al.* as per the Scopus database. The early articles were mainly focused on the corrosion mechanism. While recent citing articles were starting to elucidate some approaches to reduce the corrosion effect through material improvement^180^ and surface engineering.^181,182^ Schematic development of the hotspot (F) can be seen in Fig. 11.

### 3.2 Anticipated future research trend

The timeline visualization in Fig. 2 indicates that the most active cluster in the hotspot (A) is cluster ID #1. In this particular hotspot, Loghman-Estarki M. R. *et al.*^183^ and Ozgurluk Y. *et al.*,^71^ both of which are included in cluster ID#1, have both shown citations bursts for their articles in recent years (see Table 3). These articles focused primarily on TBC development. The majority of recent research articles citing Loghman-Estarki M. R.'s work have focused on TBC structure engineering, such as the development of multilayer TBC coatings,^184,185^ and nanostructure TBC coatings.^21,186^ Interestingly, due to the promising thermo-physical properties of high-entropy ceramics (HECs), the citing article by Xue Y. *et al.*^187^ developed a thermal barrier material based on HECs that has the composition of (La_0.2_Nd_0.2_Sm_0.2_Eu_0.2_Gd_0.2_)_2_Ce_2_O_7_ or also called HECO. Thus, the anticipated research trend may dominantly focus on TBC structure engineering, such as multilayer structure, nanoscale structure, and the development of HEC based TBC.

In the hotspot (B), burst analysis suggested that the active citation indicated by the work of Salehnasab B. *et al.*,^86^ Xuebin Wang *et al.*,^188^ and Ulrich Kleinhans *et al.*^84^ All these three active research articles were included in cluster ID#2. Xuebin Wang and Ulrich Kleinhans work mainly discussed ash formation. Recent citing articles of those works still try to elucidate the ash formation mechanisms.^189–191^ Hence, the topic of ash or deposit formation may still attract some researcher attention in the near future.

Nonetheless, the timeline visualization in Fig. 2 suggests that the active research for hotspot (B) is also indicated from cluster ID#6. Members in cluster ID#6 mainly discuss the development of materials and metal coatings to improve the hot corrosion resistance against molten salts and chloride containing molten salts. The most cited article in this cluster was published by Jie Cai *et al.*,^192^ which discussed CoCrAlY coating behavior on hot corrosion. However, based on Tables 4–6, most of the coating material was dominantly based on nickel with several developed methods of deposition and structure modification. Thus, it can mean that the direction of future research for the hotspot (B) will also cover the topic of metal coatings.

Cluster ID#3 is the only member of the hotspot (C). Based on the timeline visualization of the clusters and their corresponding hotspots in Fig. 2, hotspot (C) has not attracted much attention recently. However, two main discussions are possibly derived based on the burst analysis, as seen in Fig. 6. First is the measure to decrease the aggressiveness of the chloride through sulfidation, additive utilization, and fuel pre-treatment. Second is materials and coatings improvement to mitigate the hot corrosion. Notably, the second main discussion burst article by Oksa M. *et al.*^123^ continues to receive active citations. Thus, for the hotspot (C), the materials and coatings topic will have more chance to develop in the near future. Nevertheless, some discussion and reference co-citation with the hotspot (B) would be overlapping.

For hotspots (D) and (E), timeline visualization showed that these hotspots had no active discussion in recent years. Hence, it is rather complicated to asses possible research direction in the near future on the topic discussed by those hotspots. However, the works in the cluster member of those hotspots have built the intellectual base on the related topic. For instance, though the topic is not quite attractive, the burst work article from Fleig D. *et al.* in the hotspot (D) has inspired citing articles^190,193^ to bring the discussion to a new level of SO_2_ reducing methods and their efficiency. Similarly, citation burst work in the hotspot (E) by Skrifvars *et al.*^161^ has contributed significantly to the development of coatings and materials against hot corrosion under deposit. Therefore, recent citing articles of Skrifvar's work may have overlapping citations with articles members of the hotspot (B). As a result, the discussion in hotspot (E) may eventually lead to an issue analogous to the one in the hotspot (B) in the future direction.

On the other hand, hotspot (F) in timeline visualization is still active in recent years. The strongest burst resulted from the work from Yun-Gang Wang *et al.*^179^ that discussed the mechanism of the coupling effect between dew point corrosion and ash deposition. However, some recent citing articles discuss corrosion problems and mitigation through coatings and materials development.^180,181,194,195^ Therefore, the future trend direction is likely focused on the coating and materials improvement research for combating dew point corrosion.

## 4 Conclusions

A map of co-cited network by ten clustering labels was obtained. By utilizing the log-likelihood ratio (LLR) algorithm, the clustering labels were generated using the most representative term from the article's member. The clusters were then grouped into six research hotspots and denoted with hotspots (A), (B), (C), (D), (E), and (F). Each of the hotspots has the main topic discussion of TBC materials and their performance under molten salts exposure; materials and coatings for chloride containing molten salt environment; materials and coatings under the influence of aggressive environment; flue gas fate in the boiler; and investigations on the deposit that may induce hot corrosion in the boiler respectively.

Burst analysis of those hotspots derives an intellectual base of the hot corrosion research. Then, it leads to more narrowed discussion topics. They include discussions on the mechanisms which involve the rapid dissolution of material elements by molten salt eutectic; studies about the role of some corrosive elements like oxygen and chlorine; the works examine ash's formation and deposition in combustion systems that use coal and biomass; eventually, the discussion about materials and coatings development to mitigate the hot corrosion. These topics have intrigued researchers in almost every hotspot, including recent citing articles.

Based on those discussion topics, the future trends of the specific hot corrosion research topics are possibly elucidated. The first anticipated topics will likely focus on TBC structure engineering, including the development of multilayer and nanoscale structures and HEC-based TBCs. Then, the topic of ash or deposit formation may continue to be a research focus in the near future. Finally, methods of hot corrosion mitigation through the development of coatings and materials will likely be notable to many researchers in the hot corrosion research field.

## Author contributions

Andrieanto Nurrochman: conceptualization, methodology, formal analysis, data curation, investigation, writing – original draft. Endro Junianto: resources, validation. Akhmad Ardian Korda: supervision, validation, writing – review & editing. Budi Prawara: supervision, resources, validation, funding acquisition, writing – review & editing. Eddy Agus Basuki: supervision, conceptualization, methodology, formal analysis, project administration, validation, writing – review & editing.

## Conflicts of interest

There are no conflicts to declare.

## Supplementary Material
